# Correlation between serum cytokines and clinicopathological features in patients with drug-induced liver injury

**DOI:** 10.3389/fphar.2022.1070802

**Published:** 2022-12-08

**Authors:** Yu Zhang, Hui Gao, Yu Zhang, Yue-Ming Shao, Rui-Hua Zhang, Xiao-Yu Wen

**Affiliations:** ^1^ Department of Pediatric Endocrinology, Genetics and Metabolism, First Hospital of Jilin University, Changchun, Jilin, China; ^2^ Department of Hepatology, Shandong Provincial Third Hospital, Jinan, Shandong, China; ^3^ Department of Hepatology, First Hospital of Jilin University, Changchun, Jilin, China; ^4^ Center of Infectious Diseases and Pathogen Biology, First Hospital of Jilin University, Changchun, Jilin, China

**Keywords:** drug-induced liver injury, cytokines, clinicopathological features, tissue inhibitor of metalloproteinases-1, chemokine C-C motif ligand 1

## Abstract

**Objectives:** Changes in serum levels of cytokines have been proposed as possible biological markers of tissue damage, including drug-induced liver injury (DILI). Here, we aimed to screen cytokine markers that have guiding significance for the degree of inflammation of DILI.

**Patients and methods:** 54 patients with DILI were retrospectively analyzed as the experimental group, and 14 healthy subjects were randomly selected as the control group. A total of 20 cytokines were detected by using a cytokine protein antibody chip, and differentially expressed proteins were screened.

**Results:** There were significant differences in serum cytokines between DILI patients and healthy controls. Compared with the control group, the DILI group expressed 11 differential proteins. IL-8, TNF RII, TNFα, TNF RI, MIP-1β, MIP-1α, and IL-1β were differentially expressed in DILI patients with different degrees of inflammation from G1 to G4. MIG, IL-12p40, and IL-10 were differentially expressed in the higher degree of inflammation groups (G2, G3, and G4 groups). Tissue inhibitor of metalloproteinases-1 (TIMP-1) was differentially expressed in the group with the highest inflammation degree (G4 group). Chemokine C-C motif ligand 1 (I-309) was only differentially expressed in the lowest inflammation group (G1 group).

**Conclusion:** The changes and differential expression of specific cytokine levels were helpful for evaluating different degrees of inflammation of DILI.

## 1 Introduction

Drug-induced liver injury (DILI) refers to abnormal liver function caused by drugs and their metabolites, or by food additives or chemical agents (Shen et al., 2019). DILI is an exclusion diagnosis, that needs to exclude the influence of viruses, alcohol, autoimmunity, and other pathogenic factors. Changes in biochemical indices such as alanine aminotransferase (ALT), aspartate aminotransferase (AST), alkaline phosphatase (ALP), γ-glutamyl transpeptidase (GGT), and total bilirubin (TBil) are helpful for assessing the severity and prognosis of DILI. Serum biomarkers are another powerful diagnostic tool in the conventional clinical setting, where they are widely used for early detection of injury and evaluation of severity and prognosis. Cytokines, as small molecular proteins produced and secreted by cells after activation, have certain biological activities and play a significant role in the progression of DILI (Amanzada et al., 2014; Ali et al., 2020). However, the relationship between cytokines and DILI is not fully understood. This study analyzed the clinical and pathological characteristics of DILI, screened the differentially expressed proteins (DEPs) that can evaluate the degree of inflammation of DILI, and predicted and analyzed the severity of DILI in a non-invasive way.

## 2 Materials and methods

### 2.1 Patients

From March 2016 to August 2020, 54 patients with DILI who were diagnosed by liver biopsy and had a clear history of drug history or exposure to environmental poisons in the Department of Hepatology at the First Hospital of Jilin University were retrospectively analyzed as the experimental group (patients were marked with serial numbers D1 to D54), and 14 healthy subjects with normal liver function and without history of liver disease were randomly selected as the control group (N group, subjects were marked with serial numbers N1 to N14). The study was approved by the Ethics Committee of First Hospital of Jilin University and performed in accordance with the Declaration of Helsinki.

#### 2.1.1 Inclusion criteria

1) Age ≥ 18 years old; 2) Underwent liver biopsy; 3) Had a clear history of drug history or exposure to environmental poisons.

#### 2.1.2 Exclusion criteria

Viral hepatitis, alcoholic liver disease, non-alcoholic fatty liver disease, autoimmune liver disease, genetic metabolic liver disease, no drug-induced cholestatic liver disease, DILI overlaps autoimmune liver disease, and organ transplantation.

### 2.2 Methods

The general data, biochemical indices and histological examination of 54 patients were collected, and the clinical and pathological features were analyzed.

#### 2.2.1 Clinical classification of drug-induced liver injury

According to the Chinese guidelines for the management of drug-induced liver injury in 2015 (Study of Drug Induced Liver Disease of Chinese Medical Association, 2015), DILI is divided into hepatocellular type, cholestasis type and mixed type. The division standard is based on *R*-value [(ALT/the upper limit of normal (ULN))/(ALP/ULN)]. The hepatocellular type: ALT ≥ 3 × ULN and *R* ≥ 5; The cholestasis type: ALP ≥ 2 × ULN and *R* ≤ 2; The mixed type: ALT ≥ 3 × ULN, ALP ≥ 2 × ULN and 2 < *R* < 5.

#### 2.2.2 Roussel uclaf causality assessment method (RUCAM)

Extremely likely: >8 points; High probability: 6–8 points; possible: 3–5 points; less likely: 1–2 points; exclusion: ≤0.

#### 2.2.3 The grade of liver inflammatory

All the subjects underwent liver biopsy with 16G biopsy needle under ultrasound guidance and signed the informed consent before operation. The portal/periportal activity and lobular activity were used to classify the grade of liver inflammation in each sample (Batts and Ludwig, 1995). G0: None; G1: Minimal portal inflammation and lobular inflammation without necrosis; G2: Mild piecemeal portal/periportal necrosis and focal lobular necrosis or acidophil bodies; G3: Moderate piecemeal portal/periportal necrosis and severe focal necrosis of liver cells; G4: Sever piecemeal portal/periportal necrosis and Confluent necrosis includes bridging necrosis. According to the grade of liver inflammatory activity, 54 patients were divided into the G1 group, G2 group, G3 group, and G4 group (the degree of inflammation increased in turn, and no patients had grade 0 inflammation), and the correlation between cytokines and the degree of inflammation in DILI was analyzed.

#### 2.2.4 Detection of cytokines

Serum samples of 54 DILI patients and 14 healthy controls from the specimen bank were collected. Human specimens were obtained from the Department of Biobank, Division of Clinical Research, The first hospital of Jilin University. A total of 20 cytokines (G-CSF, I-309, IL-1β, IL-1Ra, sIL-6R, IL-8, IL-10, IL-12p40, MCP-1, MIG, MIP-1α, MIP-1β, TNFα, RANTES, TIMP-2, TIMP-1, ICAM-1, MIP-1d, TNF RI, TNF RII) were detected by using cytokine protein antibody chip, and DEPs were screened.

#### 2.2.5 Statistical analysis

SPSS Statistics 23.0 software and R software (version 4.1.2) were used for statistical analysis. The measurement data conforming to normal distribution were expressed in mean differences ± standard deviation. The comparison between the two groups was analyzed by independent sample *t*-test, and comparisons among three or more groups were performed using analysis of variance. The measurement data that do not conform to the normal distribution were expressed by the median (interquartile interval). The comparison between the two groups was conducted by Mann Whitney *U* test, and the comparisons among three or more groups were analyzed by Kruskal Wallis H test. The categorical data was described as number (%), and the chi-square test or Fisher exact test was used for the comparison between groups. A *p*-value less than 0.05 was considered significant. For cytokine-related data analysis, the original data were analyzed using R software analysis. DEPs were defined as those with adjusted *p*-value < 0.05, and fold change (FC)1.2 or <0.83 (absolute logFC > 0.263). Scatter plots were made by using GraphPad Prism software (version 7.0).

## 3 Results

### 3.1 Clinical features of drug-induced liver injury

In this study, there were 11 males with an average age of 48.18 ± 13.82 years and 43 females with an average age of 50.74 ± 9.68 years. In the RUCAM score, there were 3 cases with more than 8 points, 35 cases with 6–8 points, 16 cases with 3–5 points, and no patients with 1–2 points or less than 0 points. Suspicious drugs included traditional Chinese medicine (66.67%), antimicrobial drugs (7.4%), healthcare products (5.56%), non-steroidal anti-inflammatory drugs (5.56%), contact environmental toxicants (3.70%), antirheumatic drugs (3.70%), and antithyroid drugs (3.70%). One patient took Mongolian medicine, and one patient used Danazol. The clinical manifestations were jaundice in 29 patients (53.70%), fatigue in 17 patients (31.48%), abdominal discomfort in 11 patients (20.37%), gastrointestinal symptoms in 10 patients (18.52%), and pruritus in 4 patients (7.40%), and 19 patients (35.19%) went to the doctor because of abnormal liver function found in physical examination without feeling discomfort. According to the *R*-value [(ALT/ULN)/(ALP/ULN)], they can be divided into three groups: hepatocellular type, mixed type and cholestasis type, and the biochemical indices of patients were analyzed between groups. Among them, 28 cases were hepatocellular type, 13 cases were cholestasis type and 13 cases were mixed type. The correlation between the types of liver injury and biochemical indices is shown in Table 1. There were significant differences in AST, ALT, and GGT among the three clinical types (*p* < 0.05).

**TABLE 1:** Analysis of liver tests among three types of DILI.

	Hepatocellular type (*n* = 28)	Cholestasis type (*n* = 13)	Mixed type (*n* = 13)	*p-*value
AST (U/L)	322.1 (149.20, 659.00)	60.90 (32.85, 134.05)	98.20 (58.70, 395.10)	<0.001
ALT (U/L)	430.80 (266.50, 752.40)	57.1 (39.25, 82.95)	142.50 (101.50, 220.40)	<0.001
GGT (U/L)	152.10 (77.20, 343.90)	119.80 (50.05, 255.7)	222.40 (166.40, 437.40)	0.043
CHE (U/L)	6264.00 (5747.00, 7038.00)	6768.00 (4389.5, 8419.00)	6490.00 (5132.50, 7570.50)	0.938
ALP (U/L)	119.50 (92.10, 150.70)	145.5 (88.70, 201.50)	142.20 (101.65, 253.05)	0.326
TBiL (umol/L)	84.10 (19.00, 255.70)	24.80 (11.80, 189.05)	76.00 (15.40, 111.65)	0.333
DBiL (umol/L)	56.70 (8.50, 162.60)	11.00 (3.85, 132.20)	56.20 (5.80, 88.20)	0.387
IBiL (umol/L)	27.40 (12.10, 88.90)	13.80 (6.90, 56.85)	18.10 (11.00, 36.25)	0.302
TBA (umol/L)	128.40 (15.65, 241.10)	29.50 (7.10, 237.50)	38.90 (12.10, 261.35)	0.910
ALB (g/L)	37.54 ± 3.75	36.31 ± 2.68	38.24 ± 3.92	0.362

CHE, cholinesterase; DBiL, direct bilirubin; IBiL, indirect bilirubin; ALB, albumin; TBA, total bile acid.

### 3.2 Pathological features of drug-induced liver injury

All the patients underwent liver biopsy. The main pathological types are shown in Table 2. The pathological manifestations of DILI were non-specific. The main pathological features of the hepatocellular type were interfacial inflammation, focal necrosis and watery degeneration of hepatocytes and proliferation of the bile duct. The main manifestations of the cholestatic type were cholestasis and pigmentation in hepatocytes and bile capillaries, watery degeneration and mild hepatocyte injury. The main manifestations of the mixed type were interfacial inflammation, proliferation of bile ducts, and moderate or severe hepatocyte injury (including focal necrosis, fusion necrosis, and bridging necrosis) with cholestasis. In the three clinical classifications, the main manifestation of steatosis was vesicular steatosis. Among the 54 patients, there were 17 patients with grade G1, 12 patients with grade G2, 15 patients with grade G3, 10 patients with grade G4, and no patients with grade G0.

**TABLE 2:** Pathological characteristics of liver biopsy in patients with DILI.

	Hepatocellular type (*n* = 28)	Cholestasis type (*n* = 13)	Mixed type (*n* = 13)	*p*-value
Hydropic degeneration N (%)	26 (92.9)	12 (92.3)	10 (76.9)	0.396
Focal necrosis N (%)	27 (96.4)	9 (69.2)	13 (100)	0.019
Confluent necrosis N (%)	0 (0.0)	0 (0.0)	1 (7.7)	0.481
Bridging necrosis N (%)	8 (28.6)	2 (15.4)	1 (7.7)	0.298
Granuloma N (%)	0 (0.0)	1 (7.7)	1 (7.7)	0.227
Apoptosis body N (%)	1 (3.6)	1 (7.7)	0 (0.0)	0.763
Hepatocyte rosettes N (%)	2 (7.1)	0 (0.0)	1 (7.7)	1.000
Interface inflammation N (%)	21 (75.0)	3 (23.1)	10 (76.9)	0.004
Bile duct proliferation N (%)	20 (71.4)	7 (53.8)	10 (76.9)	0.503
Inflammatory cells in the portal area				
Mixed inflammatory cell infiltration N (%)	14 (50.0)	4 (30.8)	6 (46.2)	0.585
Neutrophil infiltration N (%)	1 (3.6)	0 (0.0)	1 (7.7)	0.736
Eosinophil infiltration N (%)	4 (14.3)	3 (23.1)	2 (15.4)	0.888
Hepatocyte steatosis				
Macrovesicular steatosis N (%)	1 (3.6)	1 (7.7)	1 (7.7)	0.604
Microvesicular steatosis N (%)	18 (64.3)	8 (61.5)	7 (53.8)	0.932
Mixed steatosis N (%)	0 (0.0)	1 (7.7)	0 (0.0)	0.481
Cholestasis N (%)	13 (46.4)	6 (46.2)	7 (53.8)	0.937
Hepatocyte pigmentation N (%)	12 (42.9)	6 (46.2)	1 (7.7)	0.053
Fibrosis N (%)	22 (78.6)	6 (46.2)	9 (69.2)	0.142

### 3.3 Expression of serum cytokines in patients with drug-induced liver injury

**FIGURE 1:**
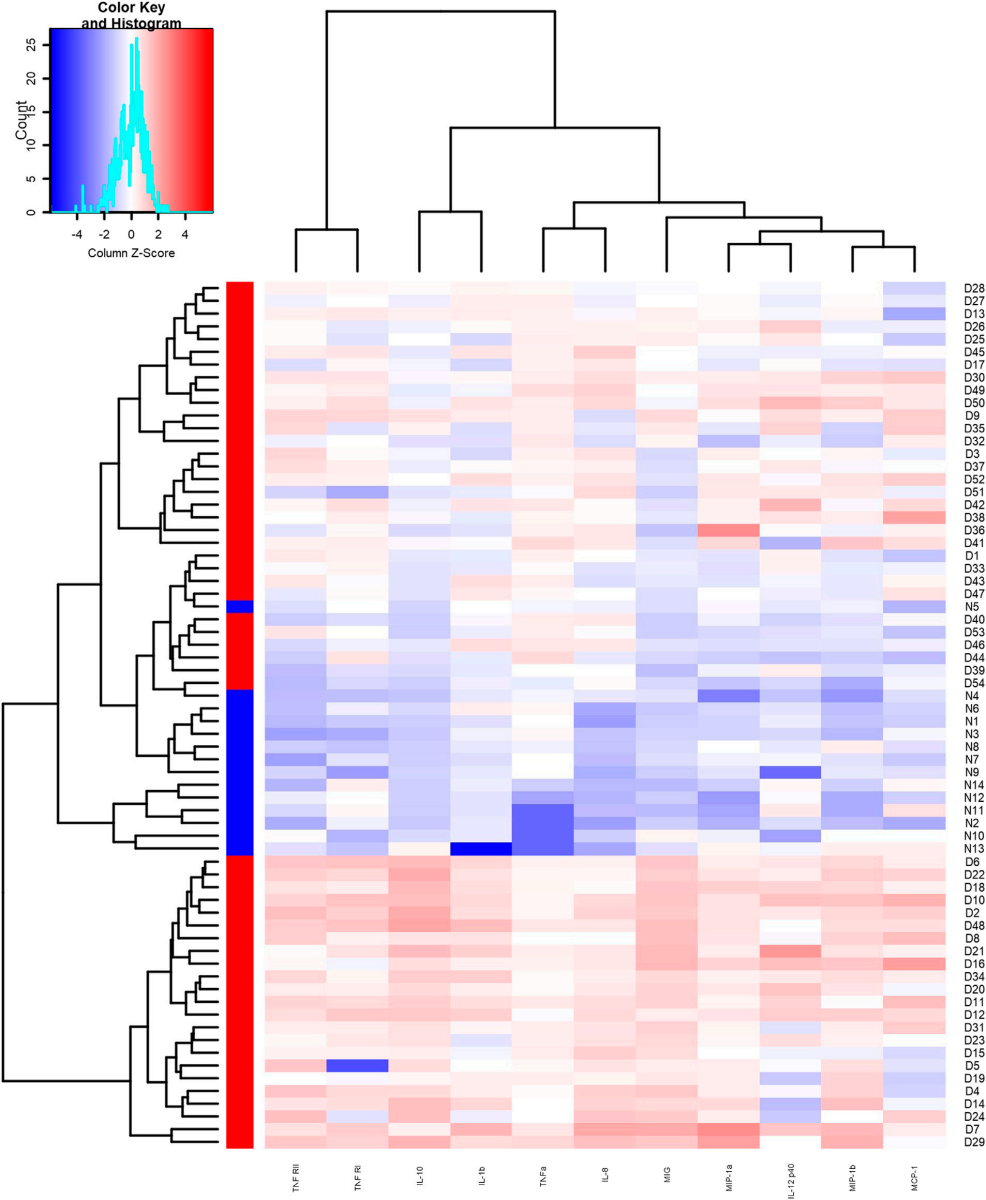
Heat map of differentially expressed proteins between the experimental group and the control group. Red area represents high expression, blue area represents low expression.

**FIGURE 2:**
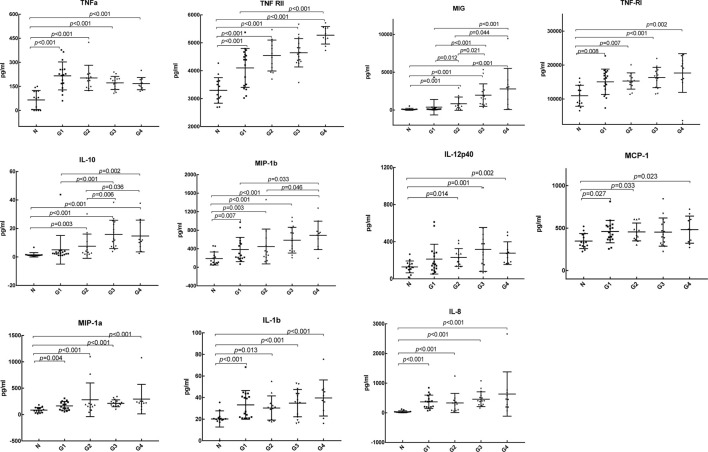
(Continued).

We detected the expression of 20 kinds of cytokines (G-CSF, I-309, IL-1β, IL-1Ra, sIL-6R, IL-8, IL-10, IL-12p40, MCP-1, MIG, MIP-1α, MIP-1β, TNFα, RANTES, TIMP-2, TIMP-1, ICAM-1, MIP-1d, TNF RI, TNF RII) in the serum of 54 patients and 14 controls. Compared with the control group, the experimental group expressed 11 differential proteins, including IL-1β, IL-8, IL-10, IL-12p40, MCP-1, MIG, MIP-1α, MIP-1β, TNFα, TNF RI, and TNF RII, which are shown in Table 3, and the expression levels were upregulated. As shown in the clustering heat map in Figure 1, 11 DEPs were highly expressed in DILI patients and expressed at low levels in healthy controls. The expression of some cytokines is shown in Figure 2.

**TABLE 3:** Differentially expressed proteins between the experimental group and the control group.

DEPs (pg/ml)	Log mean. Experimental group	Log mean. Control group	Log FC	Adj. *p*-value	Regulation
IL-8	8.340143	4.673549	3.667	<0.001	Up
TNFα	7.506853	4.387034	3.120	<0.001	Up
TNF RII	12.14067	11.67446	0.466	<0.001	Up
IL-10	2.949248	1.191627	1.758	<0.001	Up
MIP-1α	7.543348	6.136106	1.407	<0.001	Up
MIP-1β	8.706692	7.224526	1.482	<0.001	Up
MIG	9.0634	6.124296	2.939	<0.001	Up
IL-1β	5.03668	4.160451	0.876	<0.001	Up
TNF RI	13.90891	13.36666	0.542	<0.001	Up
IL-12p40	7.743396	6.771845	0.972	0.001	Up
MCP-1	8.78693	8.401937	0.385	0.006	Up

IL, interleukin; MIP, macrophage inflammatory protein; MCP, monocyte chemoattractant protein; MIG, monokine induced by interferon *γ*; TNF, tumor necrosis factor.

**FIGURE 3:**
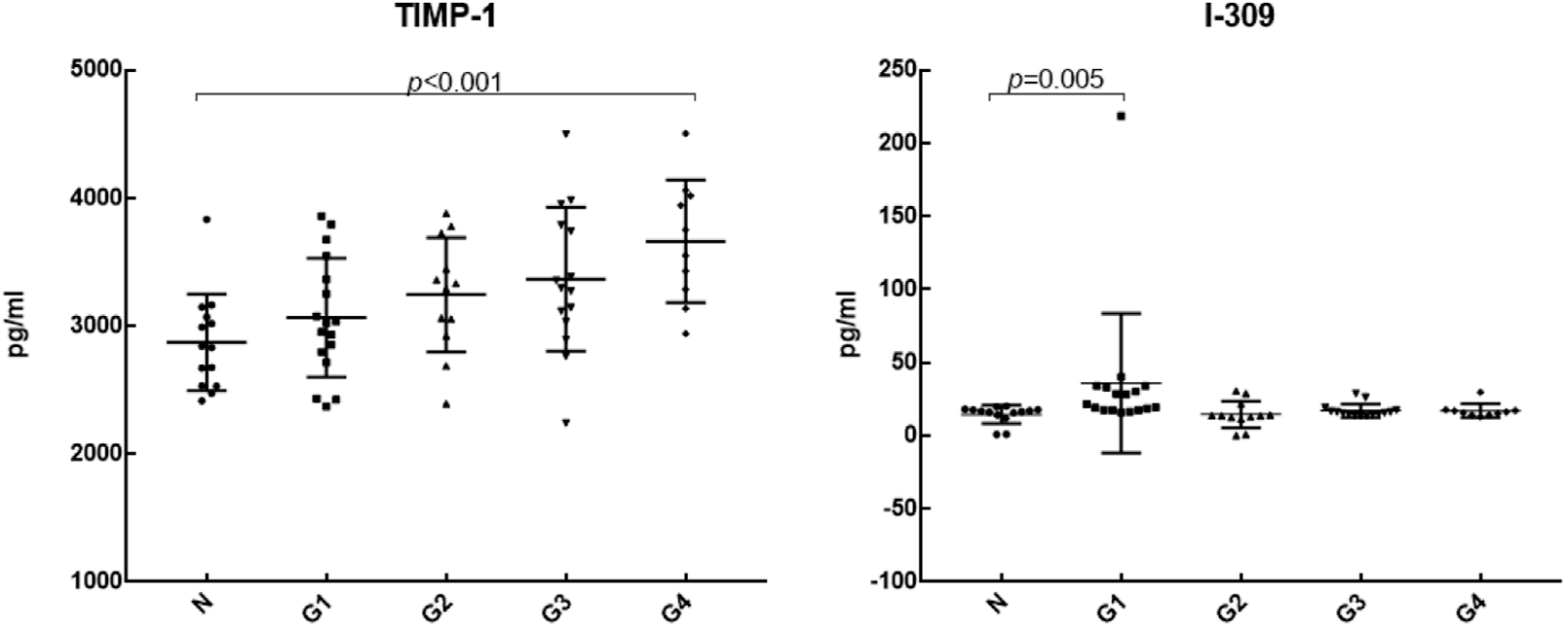
The levels of TIMP-1 and I-309 in serum.

We further analyzed the differential expression of 20 cytokines between DILI patients with different degrees of inflammation and healthy controls, as shown in Table 4. Compared with the control group, IL-8, TNF RII, TNFα, MIP-1β, MIP-1α, IL-1β, and TNF RI were differentially expressed in DILI patients with different degrees of inflammation from G1 to G4. MIG, IL-12p40, and IL-10 were differentially expressed in the higher degree of inflammation groups (G2, G3, and G4 groups) but not in the G1 group. TIMP-1 was differentially expressed in the group with the highest inflammation degree (G4 group). I-309 was only differentially expressed in the lowest inflammation group (G1 group). The levels of TIMP-1and I-309 are shown in Figure 3.

**TABLE 4:** Comparison of differentially expressed proteins between groups with different degrees of inflammation and control group.

Group	DEPs
G4 vs. N	IL-8, TNFα, TNF RII, TNF RI, MIP-1β, MIP-1α, IL-1β, MIG, IL-10, IL-12p40, TIMP-1, MCP-1
G3 vs. N	IL-8, TNFα, TNF RII, TNF RI, MIP-1β, MIP-1α, IL-1β, MIG, IL-10, IL-12p40, G-CSF
G2 vs. N	IL-8, TNFα, TNF RII, TNF RI, MIP-1β, MIP-1α, IL-1β, MIG, IL-10, IL-12p40, MIP-1d, MCP-1, G-CSF
G1 vs. N	IL-8, TNFα, TNF RII, TNF RI, MIP-1β, MIP-1α, IL-1β, I-309, MCP-1

Comparison of cytokines among different degrees of inflammation groups showed that IL-10 was differentially expressed in the G1 vs. G3 group, G1 vs. G4 group, G2 vs. G3 group, and G2 vs. G4 group. MIG was differentially expressed in the G1 vs. G2 group, G1 vs. G3 group, G1 vs. G4 group, G2 vs. G3 group, and G2 vs. G4 group. With the aggravation of inflammation, the expression level of MIG was increased.

## 4 Discussion

DILI is one of the clinical challenges due to increasing morbidity and mortality and the absence of a gold standard for diagnosis (Shen et al., 2019). The incidence rate of drug-induced liver injury is gender difference. In this study, the incidence of DILI was higher in females. Some studies suggest that the reason for this phenomenon may be related to the difference in liver enzyme metabolism, renal clearance and so on between males and females (Song et al., 2020). In addition, DILI was more likely to occur in middle-aged patients in this study, which may be related to the high social work pressure, the emergence of some basic diseases, and the increased intake of traditional Chinese medicine and healthcare products to improve general health. Both prescription and over-the-counter drugs can lead to DILI. In different countries and regions, the proportions of suspicious drugs are different. Acetaminophen is the main drug leading to liver failure in the United States (Fisher et al., 2015). In recent years, many retrospective studies have shown that traditional Chinese medicine (including Chinese herbal medicine and Chinese patent medicine) is the main cause of DILI in China (Shen et al., 2019; Song et al., 2020). In our study, traditional Chinese medicine was the main drug leading to DILI. Due to the different components and doses of traditional Chinese medicine used by each patient and the unclear interaction between components of traditional Chinese medicine, it is difficult to evaluate DILI induced by traditional Chinese medicine. Therefore, the detection of traditional Chinese medicine should be the focus of prevention and treatment of DILI in China.

DILI has no specific pathological features and can show any morphological features of acute and chronic liver diseases. Although the pathological value of DILI remains controversial, puncture pathology has important implications for the diagnosis and severity of DILI as well as the mechanism of DILI. Pathological studies on suspected DILI in DILIN (Kleiner et al., 2014) suggested that the histological features of hepatocyte injury type are more severe inflammation, necrosis and apoptosis. The histological features of cholestasis type are more often tubular and hepatocyte cholestasis (Kleiner et al., 2014). Compared with hepatocyte injury type, the histological changes of mixed type are closer to cholestasis type (Kleiner et al., 2014). In our research, the pathological features of the hepatocellular type were mainly severe lobular and portal inflammation, the histological features of the cholestasis type were mainly cholestasis and pigmentation in hepatocytes and bile capillaries, and the mixed type had the above pathological features at the same time, which was consistent with previous studies. Kleiner’s study (Kleiner et al., 2014) also suggested that some morphological changes in DILI, such as severe necrosis and microbubble steatosis, were associated with poor prognosis, and eosinophil infiltration and granuloma were associated with better prognosis. However, the sample size of our study was small, we need to expand the sample size to explore the correlation between DILI histological changes and prognosis in the future.

At present, serum markers of DILI are not ideal for judging the prognosis of DILI. The sensitivity and specificity of commonly used biomarkers such as AST, ALT, ALP are limited. Liver biopsy is an invasive procedure and its application is limited. For serious and potentially fatal DILI, it is necessary to find better biomarkers for early evaluation. Cytokines may be considered as a new biomarker of DILI. There have been many studies on the changes in single or several cytokines in DILI (Gandhi et al., 2010; Goto et al., 2015). The imbalance between anti-inflammatory cytokines and pro-inflammatory cytokines is considered to be related to the occurrence and development of DILI (Goto et al., 2015). Our previous research found that the distribution and level of cytokines in different degrees of hepatic inflammation in DILI were different. In this study, we detected and further analyzed cytokines in 54 subjects and 14 healthy controls. We found that there were significant differences in serum cytokines in patients with DILI, and the changes in several cytokines were related to the degree of inflammation of DILI.

According to the DILIN study, which was conducted in 2018 (Bonkovsky et al., 2018), when a MELD score ≥20 was used as the severity classification, the levels of IL-12, IL-17, PDGF bb, RANTES, and TNFα in DILI patients with MELD ≥20 were significantly decreased, while the levels of IL-8 and IL-6 were increased. In the study of Xie et al. (2019), the serum levels of PDGF bb, TNFα, IP-10, IL-1Ra, and MIP-1β were significantly different between severe and non-severe DILI groups. The level of TNFα was lower in the severe DILI group than that in the non-severe DILI group, but was still higher than that in the healthy control group (Xie et al., 2019). It is suggested that changes in serum-specific cytokines are associated with poor prognosis. Similarly, in our study, TNFα was higher than that in the control group, but with the aggravation of inflammation, the expression level gradually decreased, which was consistent with the above studies. It is suggested that the expression of TNFα is increased in DILI, and the decrease in TNFα may be related to the aggravation of inflammation. However, there was no statistically significant difference in the expression of TNFα among groups with different degrees of inflammation. We need to verify it in a larger sample in the future. In addition, with the increase in the degree of inflammation, the expression level of the anti-inflammatory cytokine IL-10 showed an overall upward trend, the expression level of the chemokine MIG gradually increased, and the differential expression of IL-10 and MIG in the higher degree of inflammation groups (G2, G3, G4) indicated that the increase in IL-10 and MIG levels could reflect the severity of hepatic inflammation. TIMP-1, a tissue inhibitor of matrix metalloproteinase with strong activity, is associated with liver fibrosis (Yoshiji et al., 2000; Ries, 2014). The more serious the hepatic fibrosis is, the higher the level of TIMP-1 gene expression (Nie et al., 2004). In the study of Yin et al. (2004), TIMP-1 had a good correlation with piecemeal necrosis and bridging necrosis, it suggested that TIMP-1 could not only be used as a marker for evaluating fibrosis but also had a certain value in judging the prognosis of patients with chronic liver disease. In our study, TIMP-1 was differentially expressed in the highest degree of inflammation, it suggests that TIMP-1 has value in evaluating the prognosis of DILI. But there was no significant difference in DILI patients with different fibrosis stages in our study, which may be related to the small sample size of this study. Chemokine I-309, which is closely related to Th2 cells, was only differentially expressed in the G1 group. The role and mechanism of I-309 in liver disease are less studied, and more basic studies are needed to elucidate the mechanism.

In this study, we provide insights into the association of some specific cytokines with the degree of inflammation of DILI. However, this finding should be verified in a study with a larger sample.

## Data Availability

The data of this study are available from the corresponding author upon request.
